# What constitutes disease modification in IgA nephropathy?

**DOI:** 10.1093/ckj/sfag265

**Published:** 2026-08-10

**Authors:** Nobuo Tsuboi, Osamu Hotta

**Affiliations:** Division of Nephrology and Hypertension, The Jikei University School of Medicine, Tokyo, Japan; Division of Internal Medicine, Hotta Osamu Clinic, Sendai, Japan

To the Editor,

The therapeutic landscape of immunoglobulin A (IgA) nephropathy has changed more profoundly over the past decade than at any other time in its history. Emerging immune-targeted therapies have demonstrated meaningful clinical benefits, fundamentally changing expectations for patients with IgA nephropathy [1, 2]. Collectively, these therapies target both the upstream generation of nephritogenic immune responses and the downstream mechanisms responsible for kidney injury. For the first time, these complementary therapeutic approaches have provided human proof-of-mechanism by demonstrating that selective modulation of disease-associated pathways can alter the clinical course of IgA nephropathy. At the same time, as therapeutic options continue to expand, clinicians will increasingly face questions regarding how existing and emerging therapies should be positioned, including treatment duration, how durable remission can best be maintained, and ultimately whether treatment can be safely withdrawn. This is therefore an appropriate time to step back from individual therapeutic strategies and reconsider what therapeutic success should ultimately represent in IgA nephropathy.

Current discussions of disease modification in IgA nephropathy have understandably focused on reductions in proteinuria and preservation of kidney function [3]. These remain essential therapeutic goals and robust surrogate markers of long-term outcome. However, because IgA nephropathy typically evolves over decades, these outcomes do not necessarily capture whether the underlying disease process has been fundamentally modified. The ultimate therapeutic goal is not simply disease suppression, but durable remission, ideally sustained after treatment withdrawal.

Clinical observations provide clues to the importance of immune homeostasis in IgA nephropathy. Episodes of macroscopic hematuria have long been recognized to follow upper respiratory tract infections, and more recently, new-onset or relapsing IgA nephropathy has been reported following COVID-19 vaccination [4]. Although these settings are biologically distinct, both indicate that transient disruption of immune equilibrium can unmask or amplify the same disease phenotype. These observations suggest that disruption of upstream immune regulation precedes activation of downstream pathogenic pathways. In turn, sustained remission after treatment withdrawal may ultimately depend on restoration of upstream immune homeostasis, regardless of how remission is achieved.

Indeed, restoration of such upstream immune homeostasis may occur in a subset of patients who achieve sustained clinical remission following conventional treatment. This concept is further supported by repeat-biopsy studies demonstrating histological regression together with disappearance of mesangial IgA deposits years after treatment [5]. These findings suggest that the underlying biological changes may persist well beyond the period of active therapy. While achieving remission remains a fundamental therapeutic goal, an equally important question is what biological state allows remission to persist. Addressing this question will require longitudinal studies including comparisons between patients who achieve durable treatment-free remission and those who relapse after treatment withdrawal. Such studies should aim to identify biomarkers reflecting restoration of upstream immune regulation, which may ultimately provide a common biological benchmark for evaluating therapeutic success across existing and emerging therapies. Ultimately, these insights may help guide more individualized treatment strategies.
